# Competition between Kardar–Parisi–Zhang and Berezinskii–Kosterlitz–Thouless kinetic roughening on (001) singular surface during steady crystal growth

**DOI:** 10.1038/s41598-024-79380-5

**Published:** 2024-11-29

**Authors:** Noriko Akutsu, Yoshihiro Kangawa

**Affiliations:** 1 Research Institute for Applied Mechanics, Kyushu University, 6-1 Kasuga-koen, Kasuga, Fukuoka 816-8580 Japan; 2 Faculty of Engineering, Osaka Electro-Communication University, Hatsu-cho, Neyagawa, Osaka 572-8530 Japan

**Keywords:** Nonlinear phenomena, Statistical physics, Surfaces, interfaces and thin films, Computational nanotechnology

## Abstract

Kinetic roughening of the (001) singular surface during steady crystal growth is studied on the basis of a lattice model using the Monte Carlo method. At a sufficiently low temperature, there are known to be two kinetic roughening points as the driving force for crystal growth $\Delta \mu$ increases. At a low driving force $\Delta \mu _\text{KPZ}^{(001)}$, there is the Karder–Parisi–Zhang (KPZ) roughening transition point. On the KPZ rough surface, elementary steps around islands are well defined though the surface is thermodynamically rough, with a roughness exponent $\alpha$ consistent with the KPZ universal value of 0.3869. Island-on-island structures were found to be crucial in forming the KPZ rough surface. To understand the effects of the atomical roughness of the (001) surface and the interplay of steps on long-period undulations on this surface, the dependence on the temperature *T* and driving force for crystal growth $\Delta \mu$ of surface quantities is investigated. At higher temperatures, additional Berezinskii–Kosterlitz–Thouless (BKT) rough and re-entrant KPZ regions are found for large $\Delta \mu$, where the crystal surface grows adhesively. A *T*–$\Delta \mu$ kinetic roughening diagram is also presented.

## Introduction

The “smooth” surface of a complete crystal is believed to grow by a two-dimensional (2D) nucleation process^1–6^. In the case where the surface grows in a 2D *poly-nucleation* process near equilibrium, researchers have recently clarified using the Monte Carlo method that the surface is thermodynamically rough even though it is atomically smooth^7^ and the step edges are well defined^8^. Here, thermodynamic roughness^9–11^ can be defined on the basis of the criteria at equilibrium

1 $$\begin{aligned} \text{Rough} \ \mathrm{surface: } \ W \rightarrow \infty \ \text{as } \ L \rightarrow \infty ; \quad \text{Smooth} \ \mathrm{surface: } \ W \rightarrow \mathrm{const.} \ \text{as } \ L \rightarrow \infty , \end{aligned}$$

where *L* is the linear size of the system and $W=W(L)=\sqrt{[h(\vec {x})-\langle h(\vec {x})\rangle ]^2}$, in which $h(\vec {x})$ is the height of the surface at site $\vec {x}$, and $\langle \cdot \rangle$ represents the ensemble average. This thermodynamically rough but atomically smooth surface is a newly discovered kinetically roughened surface^8^ with the Kardar–Parisi–Zang (KPZ) roughness exponent^12–14^ (KPZ rough surface). This surface also maintains a strongly anisotropic surface growth rate^8^. This fact provides guidance toward explaining strongly anisotropic growth such as that for snow^15^ in the Mullins–Sekerka instability^16^. Whether this fact is unique to the surface at sufficiently low temperatures or is universally true at various temperatures is not yet clear.

In the crystal-growth research field, a kinetically roughened surface refers to a surface that grows via an adhesive growth process^1,6,17,18^. The kinetic roughening point is designated as $\Delta \mu _{c}$, where $\Delta \mu$ is the driving force for crystal growth defined by $\Delta \mu =\mu _\text{ambient} -\mu _\text{crystal}$. Here, $\mu _\text{ambient}$ is the chemical potential of the ambient phase and $\mu _\text{crystal}$ is the chemical potential of the crystal phase. The kinetic roughening point can be defined in several ways. One definition is $G^*/{k_\text{B}T}=\pi a^2 \gamma _s^2/({k_\text{B}T}\Delta \mu _c) =1$^4,5^, where $G^*$ is the nucleation barrier for the critical nucleus, ${k_\text{B}}$ is the Boltzmann constant, *T* is the temperature, *a* is the lattice constant of a cubic lattice, and $\gamma _s$ is the step free energy per length. Cuppen et al.^19^ have proposed an alternative definition based on the observation of *n*-C_22_H_44_ crystal growth from *n*-hexane solutions^17^ and a Monte Carlo calculation reported by van Veenendaal et al.^20^. They defined $\Delta \mu _c$ as the zero point of the non-equilibrium step free energy, similar to the thermal roughening that belongs to the Brezinskii–Kosterliz–Thouless^21–23^ universality class (BKT rough surface).

In the BKT rough surface at equilibrium, $W^2(L)$ is expressed by the wavenumber *q* for slope fluctuations caused by a capillary wave as $W^2(L) =[{k_\text{B}T}/(2\pi g \sqrt{\det f^{(2)}})]\int _{1/L}^{1/a}q/(m^2+q^2) \text{d}q$^24–26^, where *g* is a geometrical factor, $\det f^{(2)}$ is the determinant of the surface stiffness tensor, and *m* is a constant. When *m* is not zero, $W^2(L)$ converges to a constant value (smooth surface); by contrast, when $m=0$, $W^2(L)$ diverges logarithmically because of the lower cut-off of the integral (the rough surface). At $T={T_\text{R}}$, $W^2(L)=(1/\pi ^2) \ln L$, where ${T_\text{R}}$ is the thermal roughening temperature. The amplitude $1/\pi ^2$ is specific to the BKT universal class^25,27–29^. The amplitude of the height–height correlation function $g(\vec {r})$ on the rough surface is twice the amplitude of $W^2(L)$.

In statistical mechanics and non-linear physics, kinetic roughening refers to KPZ roughening^30–32^. When surface width *W*(*L*) is extended to a time-dependent surface width *W*(*L*, *t*) with $h(\vec {x},t)$, the fluctuating interface often obeys the Family–Vicsek scaling function^30,31^

2 $$\begin{aligned} W(t, L) = L^{\alpha } f(t/L^z), \quad z = \alpha /\beta , \quad \text{for} \ t \rightarrow \infty , \quad W(L)\sim L^{\alpha }, \end{aligned}$$

where $\alpha$, $\beta$, and *z* are the roughness, growth, and dynamical exponents, respectively. In the non-equilibrium steady state, the surface width is characterised by the roughness exponent $\alpha$. On the basis of the symmetry principle, we derive a surface-growth KPZ equation that includes a non-linear term^12^ as

3 $$\begin{aligned} \frac{\partial h(t, \vec {x})}{\partial t}= v_0 + \nu \nabla ^2 h(t,\vec {x})+ \frac{\lambda }{2} (\nabla h(t,\vec {x}) )^2 + \eta _t \end{aligned}$$

where $v_0$ is the constant surface velocity, $\nu >0$ is a coefficient related to surface tension, $\lambda$ is a coefficient for the non-linear term, and $\eta _t$ is white noise in space and time. In the case of a 2D surface in a three-dimensional (3D) system, the values of these exponents are predicted to be $\alpha =0.3869$, $\beta = 0.2398$, and $z=1.6131$^13^.

The KPZ universality class is known to explain many non-equilibrium systems such as directed polymers and quantum systems^14,33,34^. However, in the case of crystal growth, the observed roughness exponents differ from those predicted by the KPZ model^14,31,35^, with the exception of several special surface systems^36–38^. Numerous studies have been conducted to determine why crystal surfaces do not become KPZ rough^31^. One of the reasons why KPZ behaviour cannot be observed in crystal growth is that surface diffusion occurs during growth using methods such as molecular beam epitaxy (MBE)^31,33,39–42^. In the case of epitaxial growth from the vapor phase, the Villain–Lai–Das Sarma (VLDS) class is known^41,43,44^. The exponents are $\alpha =(4-d)/3$, $\beta = (4-d)/(8+d),$ and $z=(8+d)/3$. When $d=2$, we know that $\alpha =2/3$, $\beta = 1/5$, and $z=10/3$. Notably, far from equilibrium, the Mullins–Sekerka^16^ interface instability is caused by surface or volume diffusion^2,4^. This instability leads to dendritic growth^4,45–47^ and formation of other spatiotemporal patterns^4,48–51^. These patterns are self-similar rather than self-affine.

Another reason is step bunching or macrostep formation at equilibrium and near equilibrium^52^. Because of microscopic step–step attraction, macrosteps are formed at sufficiently low temperatures at equilibrium^53–59^. For $0 \le \Delta \mu < \Delta \mu _{cr}$, the terraces and side surfaces are atomically and thermodynamically smooth, where $\Delta \mu _{cr}$ is a crossover point between 2D single and poly-nucleation processes at the lower edge of the macrostep^59^. The mean height of the macrosteps decreases as $\Delta \mu$ increases until disassembly occurs at $\Delta \mu _R$^60–62^. For $\Delta \mu _{cr}<\Delta \mu < \Delta \mu _R$, though both terraces and side surfaces are atomically smooth, *W* calculated by the Monte Carlo method diverges as $L \rightarrow \infty$. The provisional roughness exponent changes as $\alpha _J \approx 0.55 \sim 0.85$ following the shift of $\Delta \mu _R(L)$, and $\alpha _J$ converges to $\alpha = 0.60$ in the limit of $L \rightarrow \infty$^52^.

In this article, focusing on the (001) singular surface, we will clarify whether the KPZ roughening point and the “separate” crossover point to a BKT rough surface is a universal phenomenon or a phenomenon specific to the special temperature for nucleation-limited steady growth. To this end, the surface growth velocity *V* and surface width $W= W(L)$ are calculated for several temperatures using the Monte Carlo method as a non-conserved system.

The microscopic model based on the Monte Carlo simulation is a restricted solid-on-solid (RSOS) model. Here, “restricted” means that the nearest-neighbour (nn) height difference is restricted to 0 and $\pm 1$^63–65^. The RSOS model is equivalent to the 19-vertex model^66,67^. The model can be mapped to a 2D XY spin model^68^, the 1D quantum spin 1 chain model^69^, and the 1D delta function Bose gas model^70^ at equilibrium. Unfortunately, the model cannot be solved exactly using the Bethe ansatz^71^. Therefore, the Monte Carlo method is adopted so that the entropy effect is precisely taken into account. The model is more microscopic than the phase field model on a mesoscopic scale^15^ or the continuous model for crystal growth^1,2,72–74^, whereas it is a more coarse-grained model than the atomic model used in first-principles quantum calculations^75^.

## Results

### Model and calculation method

#### RSOS model

The energy of a surface with an orientation close to (001) and exhibiting (001) terrace roughness can be expressed by the discrete Hamiltonian^76^

4 $$\begin{aligned} {\mathscr {H}}_{\text{RSOS}} = {\mathscr {N}}\epsilon _{\text{surf}}+ \sum _{n,m} \epsilon {[}|h(n+1,m)-h(n,m)| +|h(n,m+1)-h(n,m)|{]} -\sum _{n,m} \Delta \mu \ h(n,m), \end{aligned}$$

where *h*(*n*, *m*) is the surface height at site (*n*, *m*) on a square lattice, ${\mathscr {N}}$ is the total number of lattice points, $\epsilon _\text{surf}$ is the surface energy per unit cell on the planar (001) surface, and $\epsilon$ is the microscopic ledge energy associated with nn interactions. The summation with respect to (*n*, *m*) is over all sites on the square lattice. The RSOS condition, meaning that the height difference between nn sites is restricted to $\{ 0, \pm 1\}$, is required implicitly. In this equation, $\Delta \mu$ is the driving force for crystal growth. To exclude diffusion effects, we assumed that the ambient phase is uniform. This assumption can be realised at the nanometre length scale near equilibrium. In the case where the ambient phase is an ideal solution, $\Delta \mu = {k_\text{B}T}\ln C/C_\text{eq}$^77^, where ${k_\text{B}}$ is the Boltzmann constant, *T* is the temperature, *C* is the concentration of the solute, and $C_\text{eq}$ is the concentration of the solute at saturation.

If the ambient phase is an ideal gas, $\Delta \mu = {k_\text{B}T}\ln P/P_\text{eq}$^78^, where *P* is the gas pressure and $P_\text{eq}$ is the gas pressure at equilibrium. Parameters $\epsilon$ and $\Delta \mu$ in the present model correspond to $\phi$ and $\Delta \mu$ in van Veenendaal et al.’s work^20^. The RSOS model is a more restricted model than the Kossel model used by van Veenendaal et al.^20^.

Because the RSOS model is a coarse-grained model used for first-principles quantum mechanical calculations, $\epsilon _\text{surf}$ and $\epsilon$ are related to the surface free energy in the atomic model that includes the entropy for lattice vibrations and distortions^75^. Thus, these variables are affected by temperature. However, the present work assumes constant values for $\epsilon _\text{surf}$ and $\epsilon$ in all of the calculations. On the other hand, the RSOS model is a microscopic model for continuous models or the mesoscopic phase field model. The step stiffness and surface stiffness in the RSOS model provide the values of the coefficient in the continuous^1,2,72–74^ or phase field model^15^.

**Fig. 1:**
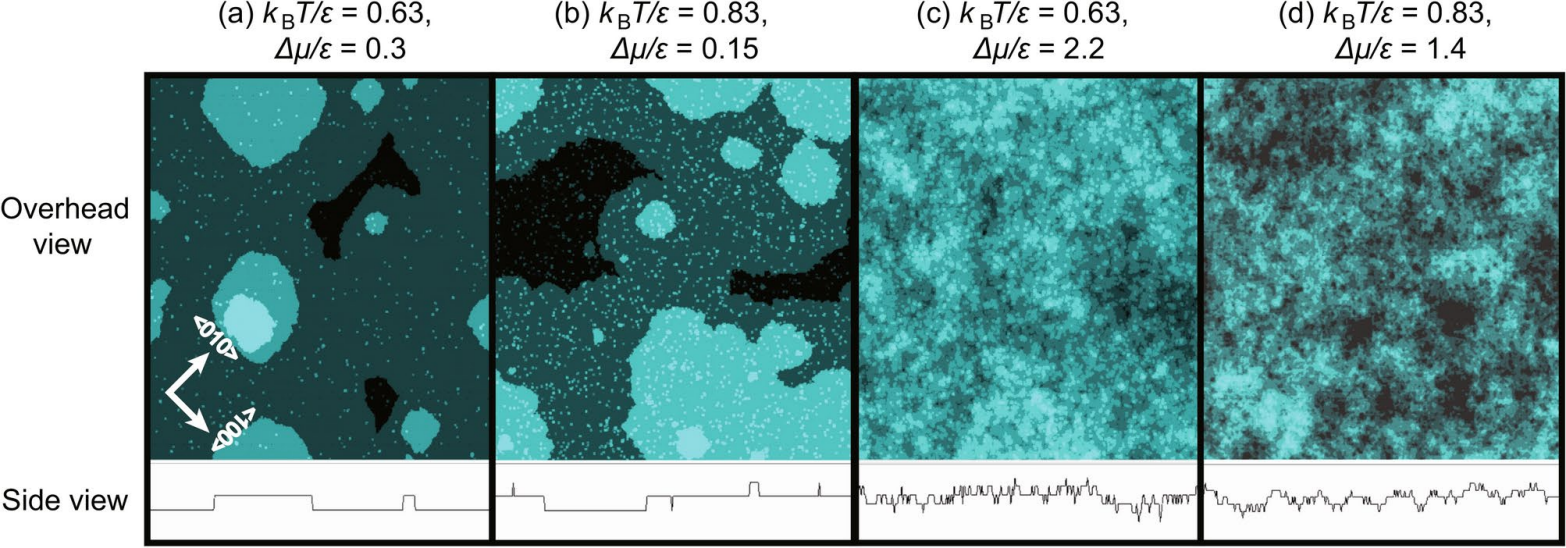
Examples of KPZ (panels (**a**) and (**b**)) and BKT (panels (**c**) and (**d**)) rough surfaces at $4 \times 10^8$ MCS/site. Upper figures show overhead views where the surface height is indicated by brightness. Lower figures show side views. In the side views, the lines indicate the surface height at the bottom line of the overhead views. $L=320 \sqrt{2}$.

**Fig. 2:**
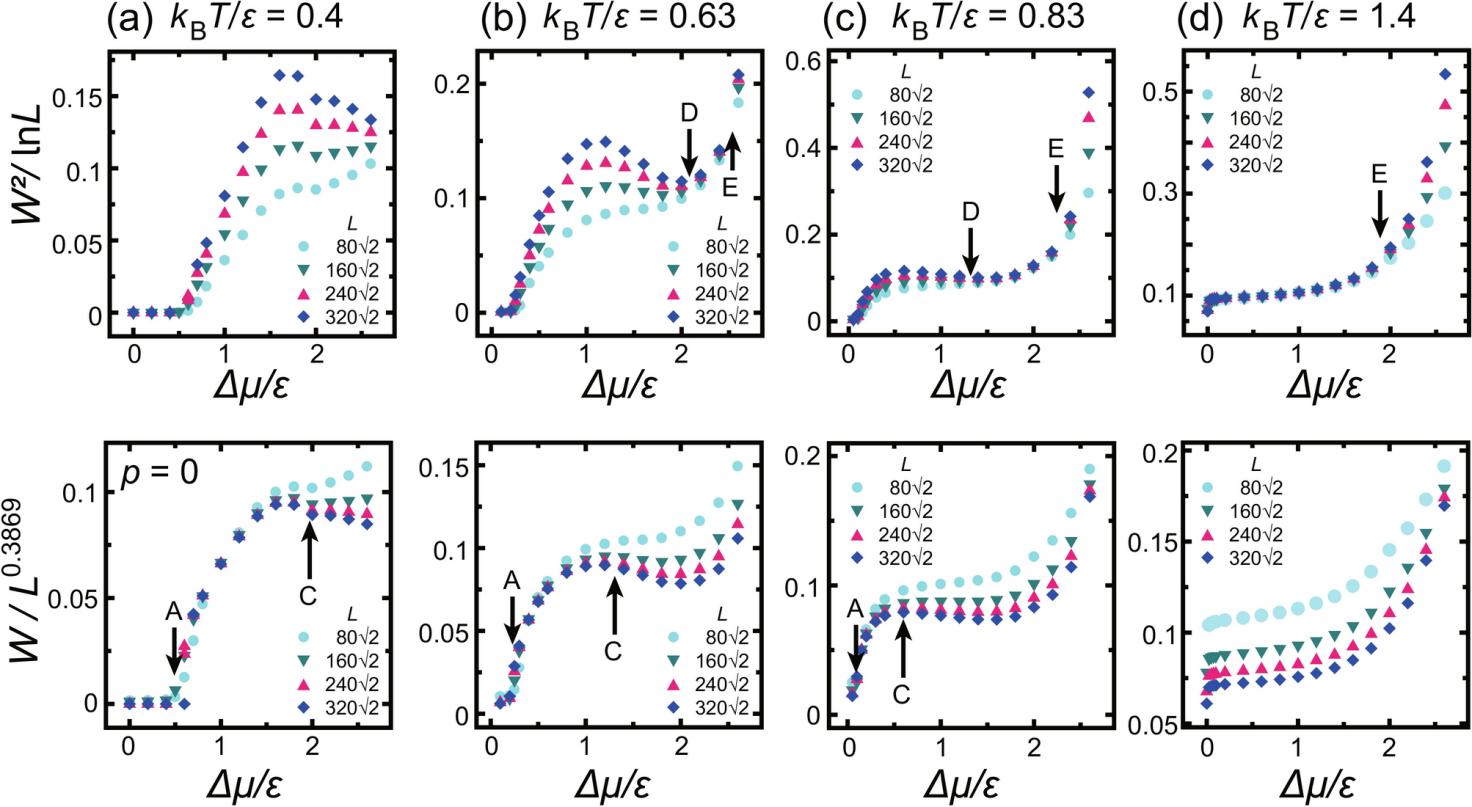
Scaled surface widths as functions of $\Delta \mu$. Upper figures: squared surface width $W^2$ scaled by $\ln L,$where *L* is the size of the system. Lower figures: surface width *W* scaled by $L^{\alpha },$where $\alpha$ is assumed to be a roughness exponent with the KPZ value (0.3869^13^). Panel (**a**) is taken from Ref.^8^ licensed under CC BY 4.0 (http://creativecommons.org/licenses/by/4.0). Panels (**b**)–(**d**) show the present numerical results. Point A: $\Delta \mu _\text{KPZ}^{(001)}$. Point C: $\Delta \mu _\text{KtoB}^{(001)}$. Point D: $\Delta \mu _\text{BKT}^{(001)}$. Point E: $\Delta \mu _\text{BtoK}^{(001)}$.

#### Monte Carlo calculations

Crystal growth proceeds by the attachment or detachment of “atoms” at randomly chosen sites, where in this work “atom” means a specific unit or cube. The surface configuration was updated using the Metropolis algorithm. The transition probability was 1 for $\Delta E= E_f-E_i \le 0$ and was $\exp (-\Delta E/{k_\text{B}T})$ for $0<\Delta E$, where $E_i$ is the surface energy of the initial configuration and $E_f$ is the surface energy of the proposed configuration. The surface energy was calculated on the basis of Eq. (4). The number of atoms in the crystal does not have to be conserved during the process, making the system a non-conserved system. The present work did not include atom exchange on the surface, i.e., surface diffusion was neglected. Note that the exponents for a fluctuating surface in a conserved system have different values than for non-conserved surfaces^79^.

The first $2 \times 10^{8}$ Monte Carlo steps per site (MCS/site) were ignored, and each quantity was averaged over the subsequent $2 \times 10^{8}$ MCS/site. After $1 \times 10^{8}$ MCS/site, the calculated values were almost constant. For several parameter sets, we averaged over the next $2 \times 10^{8}$ MCS/site ($4 \times 10^{8} \sim 6 \times 10^{8}$ MCS/site) and confirmed that the latter values agree with the former values within an error of 0.2%.

The surface slope *p* and the surface growth velocity *V* were calculated as macroscopic variables, such as

5 $$\begin{aligned} p=N_\text{step}a/L, \quad V=(\langle h(t+\tilde{\tau })\rangle -\langle h(t)\rangle )/\tilde{\tau }, \end{aligned}$$

where $N_\text{step}$ is the number of steps, which was fixed during the simulation, $a=1$ is a lattice constant, and $\tilde{\tau }$ is set to $2 \times 10^8$ MCS/site. Since the present study investigates the (001) surface of a simple cubic lattice, $N_\text{step}$ and *p* are zero.

Periodic boundary conditions were adopted in the vertical ($[\bar{1}10]$) and horizontal ([110]) directions at a rotation angle of 45 degrees around the $\langle 001 \rangle$ axis. This is to allow a comparison of the present results with the figures in Ref.^8^. In Ref.^8^, surfaces tilted towards the (111) direction were also studied, where steps run in the $\langle \bar{1}10 \rangle$ direction. Figure 1 shows an example of the simulated surfaces for several temperatures and driving forces for crystal growth.

The squared surface width was calculated as

6 $$\begin{aligned} W^2 =\langle \langle [h(\tilde{x}, \tilde{y}, t)- \langle h(\tilde{x}, t)\rangle _{\tilde{y}}]^2\rangle _{\tilde{y}} \rangle _{\tilde{x}} , \end{aligned}$$

where *W* is the surface width normal to the surface, $\tilde{x}$ and $\tilde{y}$ are the [110] and $[ \bar{1}10 ]$ directions, respectively, and $\langle \cdot \rangle _{\tilde{y}}$ and $\langle \cdot \rangle _{\tilde{x}}$ are the averages over the $\tilde{y}$ and $\tilde{x}$ directions, respectively. The calculated *W* scaled by $\ln L$ or $L^\alpha$ as a function of $\Delta \mu$ is shown in Fig. 2 for several temperatures.

Though the RSOS model is simple, there are several external parameters: *L*, *T*, $\Delta \mu$, and *p* ($N_\text{step}$). In Ref.^8^, at the fixed temperature ${k_\text{B}T}/\epsilon =0.4$, *L*, $\Delta \mu$, and *p* were systematically changed for a (001) singular surface, for which the KPZ roughening transition point $\Delta \mu ^{(001)}_\text{KPZ}$ was identified. For larger $\Delta \mu$, a KPZ rough to BKT rough crossover point $\Delta \mu ^{(001)}_\text{KtoB}$ was also found. For $\Delta \mu ^{(001)}_\text{KPZ}<\Delta \mu <\Delta \mu ^{(001)}_\text{KtoB}$, the surface fluctuation width *W* is divergent as *L* increases to $\infty$ with the KPZ roughness exponent. An analysis of the surface growth velocity *V* on the (001) surface, showed that $\Delta \mu ^{(001)}_\text{KPZ}$ agrees with the crossover point between 2D single nucleation to 2D poly-nucleation growth processes. However, whether $\Delta \mu ^{(001)}_\text{KtoB}$ can be regarded as the kinetic roughening point $\Delta \mu _c$ is not clear.

This article examines the $\Delta \mu ^{(001)}_\text{KPZ}$ and $\Delta \mu ^{(001)}_\text{KtoB}$ temperature dependence for a fixed surface slope $p=0$ (the (001) surface). For ${k_\text{B}T}/\epsilon = 0.4$, islands are formed on (001) terraces by 2D nucleation. At higher temperatures, more thermally excited islands on (001) terraces are formed and the free energy of the steps decreases. The study systematically applies various driving forces for several typical temperatures, ${k_\text{B}T}/\epsilon = 0.63$, 0.83, and 1.4, compared with the surface of ${k_\text{B}T}/\epsilon = 0.4$.

### KPZ rough surface

The driving-force dependence of the squared surface widths scaled by $\ln L$ are shown in the upper figures in Fig. 2 for several temperatures. The lower figures show the surface widths scaled by $L^{\alpha }$ with KPZ roughness exponent $\alpha$ assumed to be 0.3869. Panels (b)–(d) show the present numerical results. The surface with $0 \le \Delta \mu < \Delta \mu ^{(001)}_\text{KPZ}$ (point A in Fig. 2a–c, lower figures) has a small surface width and a constant value as the system size increases. Hence, the surface is atomically and thermodynamically smooth in addition to being at equilibrium.

For $\Delta \mu ^{(001)}_\text{KPZ}< \Delta \mu$, the surface width increases with increasing system size. Hence, the surfaces are thermodynamically rough according to the definition in Eq. (1). In particular, for $\Delta \mu ^{(001)}_\text{KPZ}< \Delta \mu < \Delta \mu ^{(001)}_\text{KtoB}$ (between points A and C in Fig. 2 (lower)), the surface width increases as $W \propto L^\alpha$, where $\alpha =0.3869$. From the Monte Carlo results, the values of $\Delta \mu ^{(001)}_\text{KtoB}$ are determined to be the largest $\Delta \mu$ for the KPZ rough region. The values of $\Delta \mu ^{(001)}_\text{KPZ}$ and $\Delta \mu ^{(001)}_\text{KtoB}$ are listed in Table 1. The KPZ roughening transition point $\Delta \mu ^{(001)}_\text{KPZ}$ decreases with increasing temperature because the step tension decreases as the temperature increases. This KPZ roughening point agrees with the crossover point between the 2D single and poly-nucleation growth modes (refer to the “Surface growth rate” subsection).

For a temperature ${k_\text{B}T}/\epsilon = 1.4$ (Fig. 2d), it is higher than the phase transition point of the 2D nn Ising model ${k_\text{B}T}_c/\epsilon = 1/\ln (1+ \sqrt{2})\approx 1.135$, whereas the temperature is lower than the thermal roughening transition temperature ${T_\text{R}}^{(001)}/\epsilon = 1.578$^56,57,66^, where ${T_\text{R}}^{(001)}$ is the thermal roughening temperature for the (001) surface. Clear $\Delta \mu ^{(001)}_\text{KPZ}$ and $\Delta \mu ^{(001)}_\text{KtoB}$ cannot be obtained.

**Table 1:** Characteristic driving forces.

	$\Delta \mu _\text{KPZ}^{(001)}/\epsilon$	$\Delta \mu _\text{poly}^{*(001)}/\epsilon$	$\Delta \mu _\text{KtoB}^{(001)}/\epsilon$	$\Delta \mu _\text{BKT}^{(001)}/\epsilon$	$\Delta \mu _\text{BtoK}^{(001)}/\epsilon$	$\Delta \mu _\text{KPZ,2}^{(001)}/\epsilon$
${k_\text{B}T}/\epsilon$	A	A′	C	D	E	F
0.4	$0.55 \pm 0.05$	$0.58 \pm 0.04$	2.00 $\pm 0.10$	>2.6	–	–
0.63	$0.25 \pm 0.03$	$0.24 \pm 0.03$	1.30 $\pm 0.10$	2.10$\pm 0.14$	2.50$\pm 0.14$	–
0.83	$0.101 \pm 0.015$	$0.06\pm 0.06$	0.6 $\pm 0.2$	1.30 $\pm 0.14$	2.30$\pm 0.14$	–
1.0	$0.03 \pm 0.03$	–	0.07 $\pm 0.07$	1.00$\pm 0.14$	2.10 $\pm 0.14$	2.70 $\pm 0.14$
1.2	–	–	–	>0	2.00 $\pm 0.14$	$2.60 \pm 0.10$
1.4	–	–	–	>0	1.90 $\pm 0.14$	>2.6

In Fig. 1a and b, the overhead and side views of the simulated surfaces for KPZ roughened surfaces are shown at ${k_\text{B}T}/\epsilon = 0.63$ and 0.83, respectively. 2D island-on-island (multilayered island) structures are clearly observed. Islands smaller than the critical nucleus are also observed and are formed more frequently than the KPZ roughened surface at ${k_\text{B}T}/\epsilon =0.4$^8^. These small islands are generated by thermal fluctuations. It is clear that the step edges around the islands are well defined. These observations indicate that the surfaces in panels (a) and (b) are atomically smooth even though they are thermodynamically rough. The surface grows via a 2D poly-nucleation process based on the well-defined surface steps. Notably, the growing surfaces are not always thermodynamically rough. For $\Delta \mu < \Delta \mu ^{(001)}_\text{KPZ}$, surfaces are atomically and thermodynamically smooth (Fig. 2).

### BKT rough surface

**Fig. 3:**
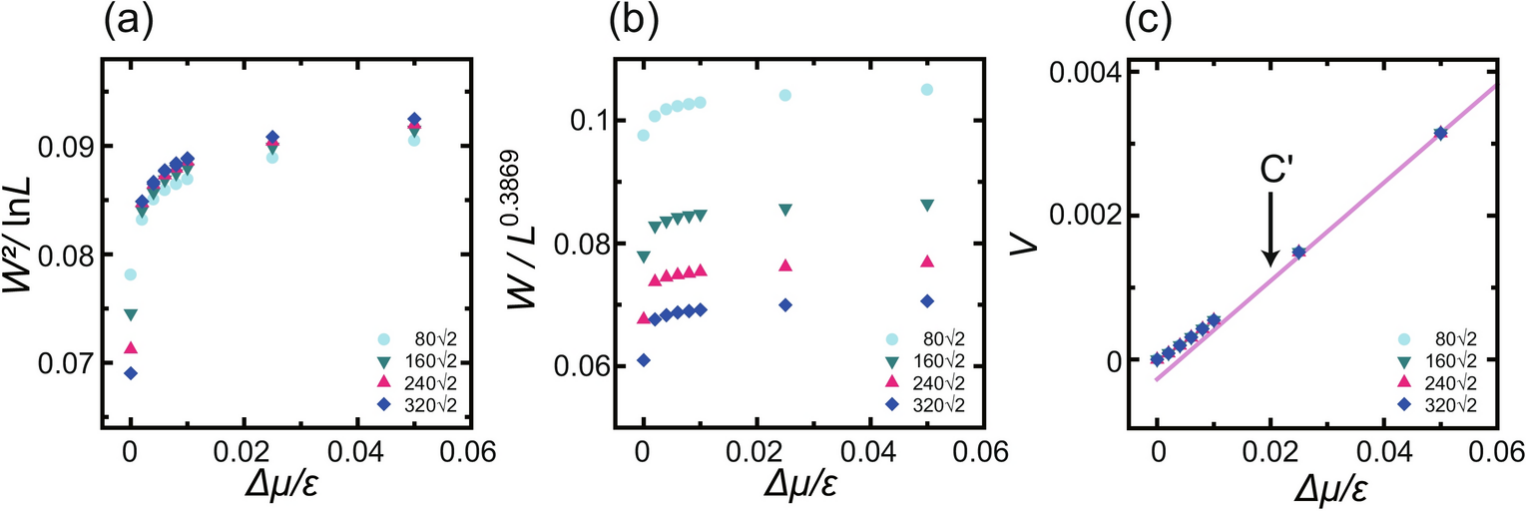
Magnified figures of scaled surface widths and surface velocity as functions of $\Delta \mu$ at ${k_\text{B}T}/\epsilon = 1.4$. (**a**) $W^2$ scaled by $\ln L$. (**b**) *W* scaled by $L^\alpha$ with $\alpha =0.3869$. (**c**) Surface growth velocity *V*. Line: $V=a\Delta \mu /\epsilon + b$ where $a=0.0684$ and $b=-2.74 \times 10^{-4}$. C′ at $\Delta \mu _{c, \text{MC}}^{(001)}$ indicates the smallest $\Delta \mu$ within the linear growth region.

In the present work, a BKT rough region has been newly found for a large $\Delta \mu$ at low temperatures. In Fig. 2 (upper figures), a BKT rough region is observed between $\Delta \mu ^{(001)}_\text{BKT}$ (points D in Fig. 2 (upper figures)) and $\Delta \mu ^{(001)}_\text{BtoK}$ (points E in Fig. 2 (upper figures)) for ${k_\text{B}T}/\epsilon =0.63$ and 0.83. For $\Delta \mu ^{(001)}_\text{BKT} \le \Delta \mu < \Delta \mu ^{(001)}_\text{BtoK}$, the $W^2$ is logarithmically divergent with *L*, similar to the thermal roughening of the BKT universality class. The values of $\Delta \mu ^{(001)}_\text{BKT}$ and $\Delta \mu ^{(001)}_\text{BtoK}$ are listed in Table 1.

In the region from point D to point E (Fig. 2 (upper figure)), the values of $W^2/\ln L$ (amplitude) are larger than the universal value of $1/\pi ^2 \sim 0.1013$, which is the amplitude at ${T_\text{R}}^{(001)}$^25,28^. The surface structures in the regions are shown in Fig. 1c and d. Small islands and negative islands (clusters of holes) are so abundant that surface steps are not discernible. Therefore, the surfaces are not only thermodynamically rough but also atomically rough except for the ramified-step region near equilibrium. The ramified-step region is the region with $\Delta \mu _\text{KtoB}^{(001)}< \Delta \mu < \Delta \mu _{c, \text{MC}}^{(001)}$, where $\Delta \mu _{c, \text{MC}}^{(001)}$ is the smallest $\Delta \mu$ in the region with a linear surface growth rate. We will discuss $\Delta \mu _{c, \text{MC}}^{(001)}$ in the next subsection “Surface growth rate”.

$\Delta \mu ^{(001)}_\text{BKT}$ decreases as the temperature increases because the population of islands and negative islands generated by thermal fluctuations increases as the temperature increases. At temperatures higher than about $T_c$ for the 2D nn Ising model, the region $0<\Delta \mu < \Delta \mu _\text{BtoK}^{(001)}$ (point E) is BKT rough. The KPZ rough region and the adjacent crossover region disappear.

At thermal equilibrium at ${k_\text{B}T}/\epsilon = 1.4$, Fig. 2d shows that $W^2/\ln L$ at $\Delta \mu = 0$ is smaller than the universal value $1/\pi ^2$ at ${T_\text{R}}^{(001)}$. Consequently, the surface is thermodynamically smooth at $\Delta \mu =0$. However, in the non-equilibrium steady state, *W* for $\Delta \mu \ne 0$ is logarithmically divergent for a wide range of $\Delta \mu$ (Fig. 2d (upper figure)). To show the details around $\Delta \mu =0$, a figure similar to Fig. 2d but with a scale magnified near $\Delta \mu =0$ is presented in Fig. 3. At equilibrium ($\Delta \mu =0$), the data in both Fig. 3a and b are scattered. As the system size increases, $W^2$ converges to a constant value. Then, $W^2/\ln L$ decreases as *L* increases (Fig. 3a). This is consistent with a thermodynamically smooth surface.

For non-equilibrium steady growth, $W^2$ increases steeply up to $\Delta \mu /\epsilon = 0.05$ and then increases gradually. $W^2/\ln L$ with different *L* agrees well for all the data in Fig. 3a except for $\Delta \mu =0$. It should be noted that $\partial V/\partial \Delta \mu$ decreases for $\Delta \mu \rightarrow 0$. This feature is the same as that for the surface growth rate with a slightly lower temperature than ${T_\text{R}}$ shown by Gilmer and Bennema^3^. The model of Gilmer and Bennema adopted a more realistic transition probability than the present model, and surface diffusion was taken into consideration. However, the features of surface growth velocity are the same as in the present study. Since the different models show the same behaviour, this phenomenon is universal and independent of the details of the system. Therefore, we conclude that the surface is BKT rough for non-equilibrium though the surface is thermodynamically smooth at equilibrium at ${k_\text{B}T}/\epsilon =1.4$.

### Surface growth rate

**Fig. 4:**
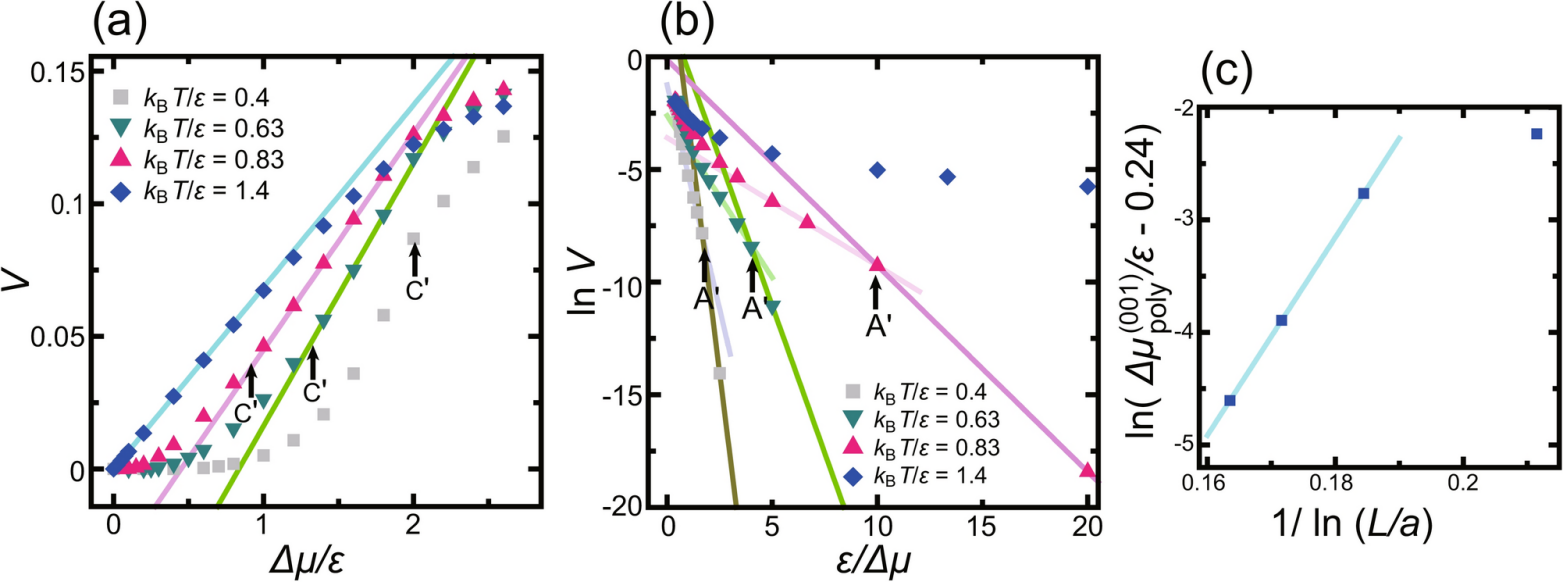
Surface growth rates for several temperatures at the (001) surface ($p=0$) as functions of $\Delta \mu$. The unit of surface growth rate is $a/\tau$, where *a* ($=1$) is the unit height and $\tau$ is the time interval for 1 MCS/site. $L=320 \sqrt{2}a$. (**a**) *V* *vs.* $\Delta \mu /\epsilon$. C′ at $\Delta \mu _{c, \text{MC}}^{(001)}$ indicates the smallest $\Delta \mu$ within the linear growth region. Lines: $V= k \Delta \mu /\epsilon -b$. Values of *k* and C′ are listed in Table 2. (**b**) $\ln V$ *vs.* $\epsilon /\Delta \mu.$ Lines: $\ln V = -g^*_\text{MC} \epsilon /\Delta \mu +b'$. Lines with lighter colour: $\ln V = -g'_\text{MC} \epsilon /\Delta \mu +b''$. Values of $g^*_\text{MC}$ and $g'_\text{MC}$ are listed in Table 2. A′ at $\Delta \mu _\text{poly}^{(001)}$ indicates the crossover point from single to poly-nucleation. (c) System size dependence of $\Delta \mu _\text{poly}^{(001)}$ at ${k_\text{B}T}/\epsilon =0.63$. Line: $\ln (\Delta \mu _\text{poly}^{(001)}/\epsilon - \Delta \mu _\text{poly}^{* (001)}/\epsilon ) = 88.0/\ln (L/a) -19.0$, where $\Delta \mu _\text{poly}^{* (001)}/\epsilon =0.24$. The converged values $\Delta \mu _\text{poly}^{* (001)}$ are listed in Table 1.

The dependence of the surface growth rate *V* on the driving force for the crystal growth, $\Delta \mu$, is shown for several temperatures in Fig. 4a. A plot of $\ln V vs. \epsilon /\Delta \mu$ is shown in Fig. 4b. The system size dependence of $\Delta \mu _\text{poly}^{(001)}$ at A′ in Fig. 4b is shown in Fig. 4c.

Near equilibrium, the surfaces grow in a 2D single nucleation process except for the surface at temperatures $1.0 {\mathop {\sim }\limits ^{<}}{k_\text{B}T}/\epsilon$. On the basis of the 2D nucleation theory^4,5,80^, the nucleation barrier for a critical nucleus $G^*/{k_\text{B}T}$ is expressed by $g^*$ as $G^*/{k_\text{B}T}=g^*/\Delta \mu$ for a single nucleation process. The values of $g^*$ obtained by the Monte Carlo method $g^*_\text{MC}$, which are the slopes of the lines in Fig. 4b, are listed in Table 2. In Table 2, the values of $g^*$ calculated using the 2D nn Ising model $g^*_\text{Ising}$ are also listed. The Monte Carlo values for ${k_\text{B}T}/\epsilon = 0.4$ refer to Ref.^8^. The Ising model values agree well with the Monte Carlo results obtained using the values in the single nucleation region.

Here, the sizes of the critical nucleus $\ell ^*$ at $\Delta \mu _\text{KPZ}^{(001)}$ are about 4, 6, and 10 for ${k_\text{B}T}/\epsilon = 0.4$, 0.63, and 0.83, respectively, based on the step free energy calculated by the 2D nn Ising model. Since $\Delta \mu _\text{KPZ}^{(001)}$ becomes small as temperature rises, $\ell ^*$ at $\Delta \mu _\text{KPZ}^{(001)}$ increases as the temperature increases. $\ell ^*$ is estimated from 1/4 of the total perimeter length of a nucleus because the shape of the nucleus is a rounded square due to the anisotropy of the step free energy.

For larger $\Delta \mu$, the nucleation mode shifts to the poly-nucleation mode^4,80^. The points designated by A′ and $\Delta \mu _{{{\text{polly}}}}^{{(001)}}$ are the crossover points between 2D single and 2D poly-nucleation. The lines for the poly-nucleation mode are shown with a lighter colour in Fig. 4b. The values of $g^{\prime}_{{{\text{MC}}}}$ are listed in Table 2. In Fig. 4b, the slope clearly changes at point A′ except for the data at ${k_\text{B}T}/\epsilon = 1.4$. Point A′ shifts to smaller $\Delta \mu$ with increasing temperature because the step tension for 2D nucleation decreases. According to the nucleation theory^4,5,80^, the slope $g'$ should be 1/3 of $g^*$; however the Monte Carlo results $g'_\text{MC}$ are approximately 1/2 of $g^*$.

The point A′ corresponds to the dynamic spinodal (DSP)^81–83^ for the 2D nn Ising model or 2D lattice gas model in the metastable state under an external field *H* or $\mu$ if the nucleation layer of the RSOS model is limited to one layer. $\phi = 4J$ and $\Delta \mu =2H$ correspond to $2\epsilon$ and $\Delta \mu$, respectively, in the RSOS model. The single nucleation region^4,80^ in crystal growth corresponds to the single droplet (SD) regime^81–83^ for small $\Delta \mu.$ The poly-nucleation region in the RSOS model approximately corresponds to the multidroplet (MD) regime. Since the crossover between single and poly-nucleation^4,80^ and the crossover between SD and MD are based on the Kolmogorov–Johnson–Mehl–Avrami (KJMA) picture, the area for nucleation shrinks as nucleation and growth proceeds. The surface growth rate decreases as time evolves. However, in the RSOS model, 2D nucleation is possible on 2D islands developed from 2D nuclei (Fig. 1a and b), which leads to steady surface growth with island-on-island structures. In the case of the 2D nn Ising model or 2D lattice gas model in the metastable state, $g'$ is known to agree with $g^*/3$^81,82^. Therefore, the discrepancies between $g'_\text{MC}$ and $g^*/3$ originate from the island-on-island structures shown in Fig. 1a and b).

The surface growth rate in the single nucleation region^4,80^ and in the SD regime^81,82^ depends on the system size *L*. Hence, $\Delta \mu _\text{poly}^{(001)}$ is expected to decrease to zero as the system size *L* increases to $\infty$. The dynamic spinodal point $\Delta \mu _\text{DSP}(T,L)$^81,82^ decreases to zero in the order of $1/\ln L$ as $L \rightarrow \infty$. However, in the RSOS model, the Monte Carlo results for $\Delta \mu _\text{poly}^{(001)}$ were found to converge to a value $\Delta \mu _\text{poly}^{* (001)}$ in the limit of $L \rightarrow \infty$. Then, we assumed $\Delta \mu _\text{poly}^{(001)}$ has the following form:

7 $$\begin{aligned} \Delta \mu _\text{poly}^{(001)}/\epsilon = \Delta \mu _\text{poly}^{* (001)} /\epsilon + c' /\ln (L/a), \end{aligned}$$

where $c'$ is a coefficient. In Fig. 4c, the *L* dependence of $\Delta \mu _\text{poly}^{(001)}$ is shown at ${k_\text{B}T}/\epsilon = 0.63$ as an example. The converged value of $\Delta \mu _\text{poly}^{* (001)}/\epsilon$ at ${k_\text{B}T}/\epsilon = 0.63$ was 0.24. In this manner, the obtained converged values $\Delta \mu _\text{poly}^{* (001)}$ are listed in Table 1. The obtained values of $\Delta \mu _\text{poly}^{* (001)}$ agree with $\Delta \mu _\text{KPZ}^{(001)}$ within the error.

At high temperatures of $1.0 {\mathop {\sim }\limits ^{<}}{k_\text{B}T}/\epsilon$, the line slope increases as $1/\Delta \mu$ decreases, similar to the case for ${k_\text{B}T}/\epsilon = 1.4$ in Fig. 4b. Hence, we cannot determine A′ for $1.0 {\mathop {\sim }\limits ^{<}}{k_\text{B}T}/\epsilon$, which is consistent with the vanishing of the KPZ rough region. The $g^*_\text{MC}$ values in Table 2 for $1.0 \le {k_\text{B}T}/\epsilon$ were estimated from the slope between points with the largest and next largest $1/\Delta \mu$ values. Since $1.0 {\mathop {\sim }\limits ^{<}}{k_\text{B}T}/\epsilon$ is around or above $T_c$ for the 2D nn Ising model, the values of $g^*$ at ${k_\text{B}T}/\epsilon =1.0$ and 1.2 are estimated with the RSOS model using the product wave-function renormalization group (PWFRG) method^84,85^, which is a transfer matrix version^86,87^ of the density matrix renormalization group^88^, or tensor network methods^89^. These $g^*$ values are designated as $g^*_\text{RSOS}$. The values of $g^*_\text{MC}$ are larger than the values of $g^*_\text{RSOS}$ calculated by the PWFRG method.

**Table 2:** Formation free energy of the 2D critical nucleus and the adhesive growth point.

	$g^*_\text{Ising}=G^*_\text{Ising}\Delta \mu /{k_\text{B}T}$	$g^*_\text{MC}$	$g'_\text{MC}$	*k*	$\Delta \mu _{c, \text{MC}}^{(001)}/\epsilon$	$\Delta \mu _c/\epsilon$	$\Delta \mu _c/\epsilon$
${k_\text{B}T}/\epsilon$	(*: $g_{{{\text{RSOS}}}}^{*}$)				C′	Ref.^19^	Ref.^5^
0.63	2.79	2.62	1.44	0.0990	1.30 $\pm 0.10$	0.962	2.79
0.83	0.903	0.914	0.568	0.0817	0.9 $\pm 0.2$	0.580	0.903
1.0	0.170*	$\approx 0.194$	–	0.0714	0.45 $\pm 0.14$	0.26	0.170
1.2	0.043*	$\approx 0.095$	–	0.0683	0.13 $\pm 0.13$	–	0.043
1.4	–	$\approx 0.037$	–	0.0684	0.02$\pm 0.02$	–	–

For larger $\Delta \mu$ values, the surface growth rate *V* in Fig. 4a changes from a concave shape to a linear shape at the special driving force indicated by point C′ in Fig. 4 for ${k_\text{B}T}/\epsilon = 0.4$, 0.63, and 0.83. The criterion used to determine point C′^8^ in the present work is the smallest $\Delta \mu$ for linear growth, which is designated as $\Delta \mu _{c, \text{MC}}^{(001)}$. The values of $\Delta \mu _{c, \text{MC}}^{(001)}$ are listed in Table 2.

**Fig. 5:**
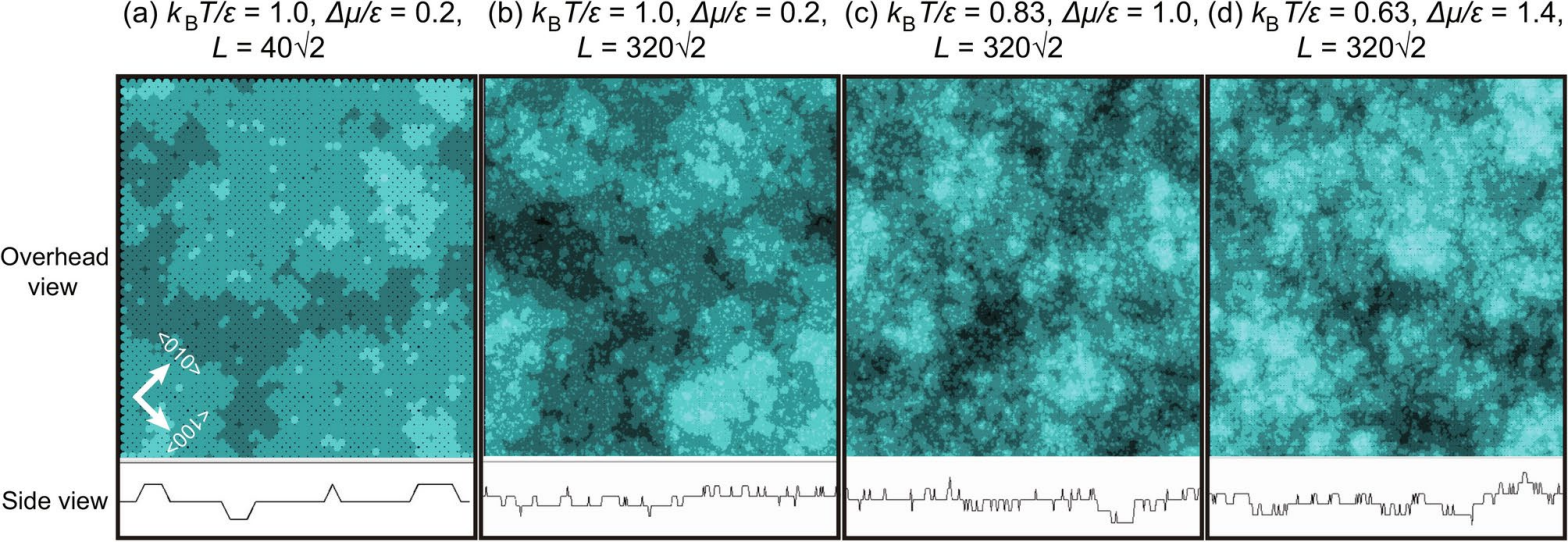
Examples of surfaces in the ramified-step region ((**a**) and (**b**)) and in the crossover region ((**c**) and (**d**)) at $4 \times 10^8$ MCS/site. Upper figures show overhead views in which the surface height is indicated by brightness. The lower figures show side views. In the side views, lines show the surface height at the bottom line of the overhead views. The height–height correlation length $\xi$ at equilibrium is 4.4*a*, 2.0*a*, and 1.3*a* for ${k_\text{B}T}/\epsilon = 1.0$, 0.83, and 0.63, respectively.

For ${k_\text{B}T}/\epsilon = 0.4$ and 0.63, the point C′ agrees with $\Delta \mu _\text{KtoB}^{(001)}$. However, C′ is not the same as $\Delta \mu _\text{KtoB}^{(001)}$ for other temperatures. For $\Delta \mu _{c, \text{MC}}^{(001)}<\Delta \mu$, the surface steps are not discernible, and the surface grows adhesively. For $\Delta \mu _\text{KtoB}^{(001)}<\Delta \mu <\Delta \mu _{c, \text{MC}}^{(001)}$, steps are discernible on the simulated surface though small clusters of adatoms and holes are frequently seen (Fig. 5a and b for example). On a short length scale, terraces can be seen and the local width^90,91^ of a step is large and islands have ramified shapes. The correlation length $\xi$ for the surface height at equilibrium at ${k_\text{B}T}/\epsilon = 1.0$ is 4.4*a* as calculated by the PWFRG method.

The ramified-step region also exists in the BKT rough region near equilibrium. As shown in Fig. 3c, *V* with small $\Delta \mu$ deviates from the line at $T_c {\mathop {\sim }\limits ^{<}}T$.

The point C′ is regarded as the kinetic roughening point $\Delta \mu _c$^1,4,5,19,20^. The surface growth rate *V* for $\Delta \mu _\text{KPZ}^{(001)} {\mathop {\sim }\limits ^{<}}\Delta \mu$ hardly depends on the system size. Thus, *V* is determined by a local structure on the surface, such as the kink density. According to the criterion that $G^*/{k_\text{B}T}=g^*/\Delta \mu _c=1$^5^, the obtained $\Delta \mu _c$ are too large (Ref.^5^ in Table 2) for ${k_\text{B}T}/\epsilon <1.0$. Cuppen et al. estimated $\Delta \mu _c$ by considering the zeros of the non-equilibrium step free energy^19,20^ (Ref.^19^ in Table 2). Disregarding whether the non-equilibrium step free energy can be well defined, the idea is similar to thermal roughening. In our results, steps are not discernible on the surfaces for $\Delta \mu _{c, \text{MC}}^{(001)} < \Delta \mu$. Qualitatively, the temperature dependence of Cuppen et al.’s $\Delta \mu _c$ describes that for $\Delta \mu _{c, \text{MC}}^{(001)}$. Quantitatively, however, Cuppen et al.’s values are small compared with the $\Delta \mu _{c, \text{MC}}^{(001)}$ values. The model of Cuppen et al. considered was the Kossel or absolute SOS (ASOS) model. Without the RSOS restriction, surface steps can fluctuate more easily than for the RSOS model. This leads to a lower thermal roughening temperature in the ASOS model than in the RSOS model at equilibrium.

The slope of the line, *k*, is expressed as $k= \partial V/\partial (\Delta \mu /\epsilon )$. The values are listed in Table 2. Contrary to expectations, *k* decreases as the temperature increases. The primary reason for this behaviour is that $\partial V/\partial (\Delta \mu /\epsilon )$ differs from $V/\Delta \mu$. At low temperatures, the absolute value of *V* is small up to relatively large $\Delta \mu$. As $\Delta \mu$ increases further, *V* increases rapidly. Consequently, $\partial V/\partial (\Delta \mu /\epsilon )$ becomes large.

### Crossover between KPZ and BKT rough surfaces

From Fig. 2, we can see that there are two crossover regions between KPZ rough and BKT rough surfaces. In the region between $\Delta \mu ^{(001)}_\text{KtoB}$ (points C) and $\Delta \mu ^{(001)}_\text{BKT}$ (points D), the surface becomes power law rough and the roughness exponent $\alpha$ appears to decrease to zero from the KPZ value as $\Delta \mu$ increases.

One reason for the crossover from KPZ rough to BKT rough surfaces originates in atomically roughening of the (001) surface. For $1<\Delta \mu /\epsilon$, where the size of a critical nucleus $\ell ^*$ becomes smaller than 2*a*^8,61^, small structures such as adatoms and clusters caused by 2D nuclei roughen the surface atomically. Eventually, the surface steps become difficult to see for $\Delta \mu _\text{KtoB}^{(001)}< \Delta \mu$ (Fig. 5c and d). In addition, thermally excited structures such as adatoms, adholes, and their clusters are created frequently at high temperatures on the (001) surface by thermal fluctuations. In off-critical regions of ${T_\text{R}}^{(001)}$, such as ${k_\text{B}T}/\epsilon = 0.63$ and 0.83, the population of small excited structures increases (Fig. 1a and b), which tends to roughen the surface atomically.

These small structures enhance or block the motion of steps. Islands or holes on a terrace with the same surface level enhance the step fluctuations to make the surface KPZ rough^64^; whereas islands or holes with a different surface level block the step fluctuations to make the surface BKT rough. For example, holes on terraces self-pin the step advancement. In this manner, the increase of atomical roughness decreases the fluctuation of steps, which tends to decrease the roughness exponent for the surface. This scenario can explain why KPZ rough surfaces cross over to BKT rough surfaces for increasing *T* and $\Delta \mu$. The increase of atomical roughness can also explain why $\Delta \mu _\text{KtoB}^{(001)}$ and $\Delta \mu _\text{BKT}^{(001)}$ become smaller as the temperature increases. It should be noted that the surface steps may not be well defined (Figs. 1c and d, and 5c and d) when the surface is sufficiently atomically rough. For such a surface, the extended step free energy^19^ may be zero. The intrinsic width of a one-dimensional interface in the 2D nn Ising model proposed by Bricmont et al.^90,91^ may be used to measure the atomical roughness for lattice systems. Evaluating the atomical roughness is a future problem to be studied.

**Fig. 6:**
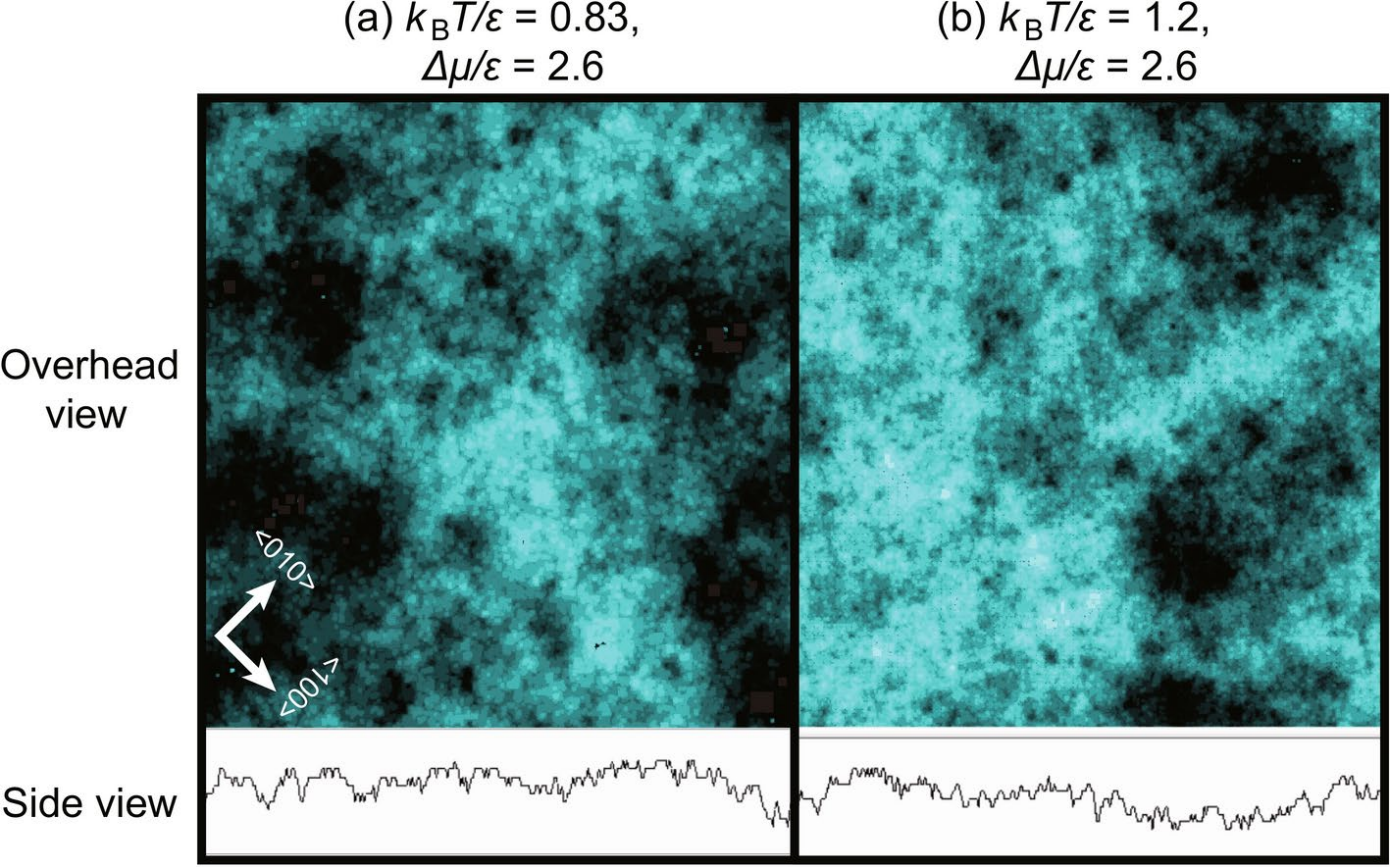
Examples of re-entrant KPZ rough surfaces at $4 \times 10^8$ MCS/site. Upper figures show overhead views where the heights are described by bright colours. Lower figures show side views. In the side views, lines show the surface height at the bottom line of the overhead views. $L=320 \sqrt{2}$.

There is another reason for crossovers from KPZ rough to BKT rough surfaces (point C and D) associated with the competition between the 2D nucleation rate and the step growth rate on the (001) surface (see the Supplementary Movie 1). Let us consider an atomically smooth (001) surface. Key parameters for the system are the mean distance between nuclei $R_0$ and mean time $t_0$ to cover one layer by the coalescence of islands or domains^80,82^. Here, we introduce another key quantity $\ell _0$, the mean distance between surface steps. When a long-wavelength undulation is formed on the surface, a surface with a slope of $p=\sqrt{p_x^2+p_y^2}$, where $p_x=\partial h/\partial x$ and $p_y=\partial h/\partial y$, is formed locally and $\ell _0$ is described by $\ell _0=a/p$. For $R_0<\ell _0$, namely a tilted surface with a sufficiently small step growth rate $v_s$, islands grown from nuclei are built on the terraces between steps and a self affine island-on-island structure tends to make the surface power law or KPZ rough locally. When the local surface slope is steep, $\ell _0<R_0$, or $v_s$ is sufficiently large, the islands on the terrace merge with the growing step before forming island-on-island structures. This suppresses surface undulation and tends to generate parallel step growth with a BKT rough surface locally. If we introduce $t_s$ as the swept time of a terrace by a step associated with the step growth rate $v_s$, we have $t_s=\ell _0/v_s$ and the relationship $R_0<\ell _0$ is replaced by $t_0<t_s.$

We can obtain $g'=g^*/2$ when $\ell _0 {\mathop {\sim }\limits ^{<}}R_0$ or $t_s {\mathop {\sim }\limits ^{<}}t_0$. 2D nuclei are assumed to appear on the terraces between steps with a step distance $\ell _0$ on the side of a multilayered island. Based on the restricted geometry picture, the 2D nucleation area is approximately $R_0 \ell _0$. Since $t_0$ is described by $t_0=1/(I_n R_0 \ell _0)$ and $t_0= R_0/(2v_s)$, where $I_n$ is the 2D nucleation rate per area as $I_n \propto \Delta \mu ^K \exp [g^*/\Delta \mu ]$^80–82^, and *K* is a kinetics dependent power, we have

8 $$\begin{aligned} R_0= \sqrt{2 v_s /(I_n \ell _0)} = \sqrt{2/(t_s I_n)}, \quad t_0 =1/\sqrt{2 v_s \ell _0 I_n} = 1/(v_s \sqrt{2 t_s I_n}), \quad V=a/t_0 = a \sqrt{2 v_s \ell _0 I_n} = a v_s \sqrt{2 t_s I_n}. \end{aligned}$$

Therefore, from the expression for $V \propto \sqrt{I_n}$, $g'=g^*/2$ is obtained, which is close to $g'_\text{MC}$.

The temperature and $\Delta \mu$ dependences of $R_0$ and $\ell _0$, or $t_0$ and $t_s$ are complex. Thus, the physical competition between $t_s$ and $t_0$, i.e., the step growth rate and 2D poly-nucleation rate on (001) terraces, drives multiple crossovers between KPZ rough and BKT rough surfaces.

There is another crossover region at even larger values of $\Delta \mu$ (points E$<\Delta \mu$ in Fig. 2). Interestingly, for $\Delta \mu ^{(001)}_\text{BtoK}$ (points E) $< \Delta \mu$, the surface width approaches that for the KPZ universality class again. In contrast to the low $\Delta \mu$ KPZ rough surface, the surface in this KPZ region is atomically and thermodynamically rough with the surface growing in an adhesive growth process. The roughness exponent $\alpha$ gradually increases from zero to the value for the KPZ universality class as $\Delta \mu$ increases. In Fig. 6, examples of the surface structures are shown for two temperatures. In addition to the small size in the up–down direction, long-period surface height fluctuations are observed.

Physically, re-entry into the KPZ rough region is probably caused by decreased detachment of atoms from the surface. The slope for linear growth changes around $\Delta \mu /\epsilon = 2$ (Fig. 4a). Assuming that the shape of a critical nucleus is square because of a finite size effect^8^, the size of the critical nucleus $\ell ^*$ equals one at $\Delta \mu /\epsilon = 2$. The phase corresponds to the mean-field spinodal (MFSP)^81,82^ for the 2D nn Ising model if the nucleation layer is limited that in the RSOS model. For $2\le \Delta \mu /\epsilon$, the atoms attached from the ambient phase onto the surface do not detach on average because $\ell ^* <1$. For the temperatures ${k_\text{B}T}/\epsilon = 0.63$, 0.83, and 1.4, the slopes of the increase of the surface growth rate *V* with respect to $\Delta \mu$ change and almost agree with each other (Fig. 4a). This result means that the growth rate reaches approximately a maximum growth rate where the atoms rarely detach. Detachment of atoms decreases to almost zero as $\Delta \mu$ increases. The surface kinetics becomes similar to that for the (2+1) Eden model with a KPZ roughness exponent^31,92^.

**Fig. 7:**
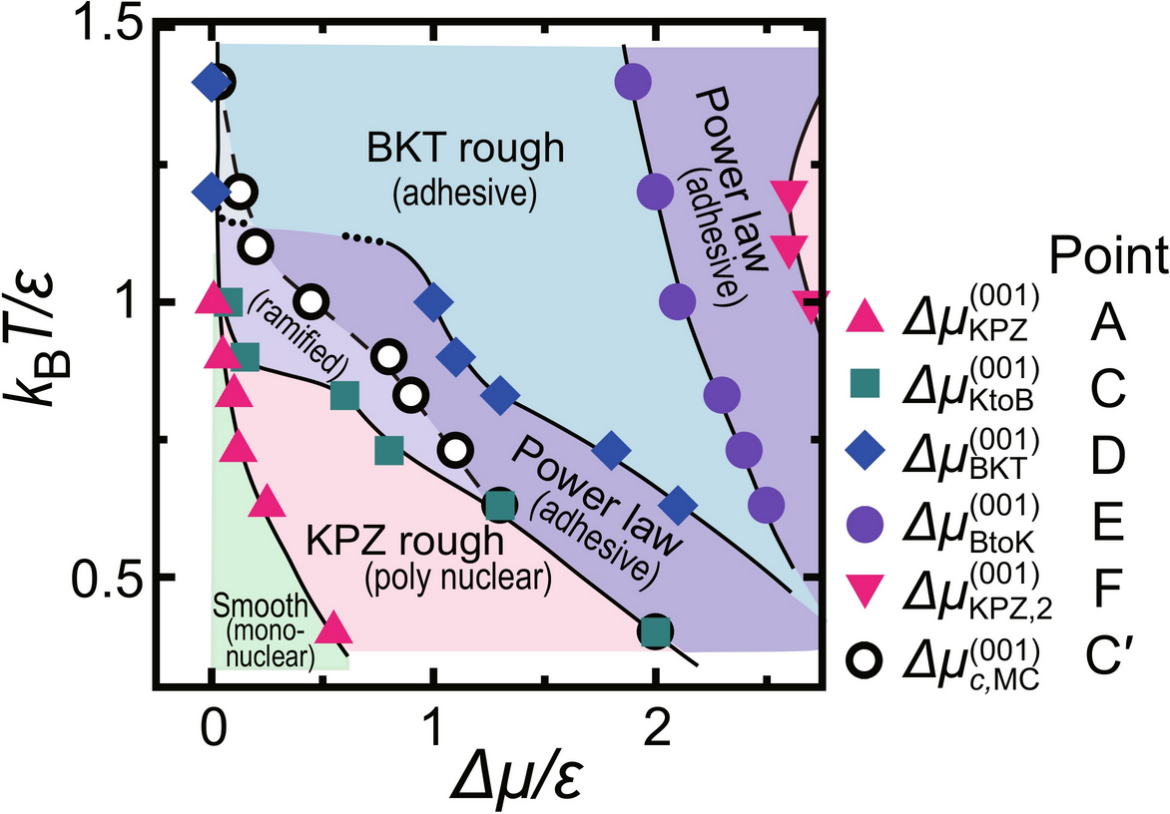
*T*–$\Delta \mu$ kinetic roughening diagram on the (001) surface.

As a summary of the kinetic roughening on the (001) surface, the *T*–$\Delta \mu$ kinetic roughening diagram is shown in Fig. 7. The upper right area coloured pale pink is the re-entrant KPZ rough region. In the “Smooth” region, the (001) surface is atomically and thermodynamically smooth.

## Discussion

Vicinal surfaces near the (001) surface at low temperatures, such as ${k_\text{B}T}/\epsilon =0.4$, were subjected to multiple crossovers between KPZ rough and BKT rough surfaces for *p* of $\Delta \mu _{cr}< \Delta \mu$^8^. It was clarified that the competition between the step growth velocity and the 2D poly-nucleation rate on (001) terraces causes the multiple crossovers between KPZ rough and BKT rough surfaces, similar to the cases on the (001) surface. Multilayered islands are frequently formed on a (001) terrace for $1< \Delta \mu /\epsilon$, which leads to a two-fold increase in *V*^8^, similar to the case in the work of Liu *et al.*^17^ or Zhang et al.^93^. To understand the multiple crossovers between KPZ rough and BKT rough surfaces including the change in surface slope, a kinetic roughening diagram will be helpful. Deriving the diagram for this case will require the calculation of a large number of parameter points in $\{ L, T, \Delta \mu , p \}$ parameter space and will be published in a future report.

2D nucleation growth in the RSOS model corresponds to the droplet theory of nucleation for the 2D nn Ising model if the nucleation layer is limited to one. The nucleation rate per area is described by $I_n \propto \Delta \mu ^K \exp [g^*/\Delta \mu ]$^81,82^ (see subsection “Crossover between KPZ and BKT rough surfaces” in “Results” section). Here, $K=3$ for the 2D Ising model^81,82^. While, $K=5/6$ for 2D nucleation and growth theory^4^, where the surface diffusion effect on steps is taken into consideration.

A new result in this work is the non-zero $\Delta \mu _\text{poly}^{* (001)}$ at low temperatures. The reason for the non-zero values is not yet clear. However, from the Monte Carlo results (Fig. 4c), the data for a large system size line up when $\Delta \mu _\text{poly}^{* (001)}$ is taken as an appropriate value, agreeing with $\Delta \mu _\text{KPZ}^{(001)}$ within the error. $\Delta \mu _\text{poly}^{* (001)}$ corresponds to $|\Delta \mu |_\text{DSP} (T,L)$ in the *d* dimensional nn Ising model with $d=2$^81,82^. At ${k_\text{B}T}/\epsilon = 0.83$, which is $0.53 {T_\text{R}}$ of the RSOS model and $0.73 T_c$ of the 2D nn Ising model, $\Delta \mu _\text{poly}^{* (001)}$ is close to zero and $\Delta \mu _\text{KPZ}^{(001)}$ also becomes small. $|\Delta \mu |_\text{DSP} (T,L)$ for the nn Ising model has typically been studied at about 0.8 $T_c$^81–83,94^. It is possible that the low temperature property may be different from that near $T_c$ in the d-dimensional nn Ising model. A future study is anticipated.

In the present work, surface diffusion, volume diffusion, and elastic effects were not considered. The chemical potentials in the ambient and crystal phases were assumed to be uniform. The aim of this work is not to reproduce realistic crystal growth, but to clarify the competition between the KPZ and BKT universal behaviours on the roughness on the (001) singular surface as the temperature changes in a minimal surface model.

KPZ behaviour in crystal growth has only recently been identified^14,31^. Our model is the first step that shows KPZ behaviour in crystal growth associated with 2D nucleation. Since both RSOS and the Metropolis algorithm^95,96^ are well-established models, it is easy to determine whether the results are intrinsic to the model or an artefact with errors when an unexpected result is obtained. Our results for *V* and the morphology of the RSOS model with the Metropolis algorithm agree well with the van Veenendaal et al. results^20^ obtained by the Monte Carlo method with the Kossel model with a kinetic Monte Carlo algorithm at low temperatures. This agreement is so close that we do not think the results for *W* are an artefact specific to the surface kinetics based on the Metropolis algorithm. The Metropolis algorithm has been used until recently to study challenging subjects such as the metastable behaviour of spin-*s* Ising and Blume-Capel ferromagnets^94^. It is expected that future studies will apply various other models to the KPZ roughness.

## Conclusions

The universality class of the kinetic surface roughness for the (001) surface changes in a complex way depending on the temperatures *T* and $\Delta \mu$ due to the competition between 1) the large period of undulation caused by multilayered islands and the small period of clusters of adatoms and holes and 2) the 2D nucleation rate and the step growth rate. The results of our Monte Carlo study of the (001) surface during interface-limited steady growth at several temperatures are summarised as follows:

- $\Delta \mu ^{(001)}_\text{KPZ}$ is the KPZ roughening transition point in non-equilibrium. For $\Delta \mu ^{(001)}_\text{KPZ}< \Delta \mu <\Delta \mu ^{(001)}_\text{KtoB}$, the surface is KPZ rough and grows continuously in the 2D poly-nucleation process at temperatures lower than the critical temperature ${k_\text{B}}T_c/\epsilon \approx 1.135$ of the 2D nn Ising model. The KPZ rough surface is atomically smooth but thermodynamically rough. $\Delta \mu ^{(001)}_\text{KPZ}$ and $\Delta \mu ^{(001)}_\text{KtoB}$ decrease as the temperature increases because the step free energy decreases. The 2D poly-nucleation rate increases drastically with decreasing step free energy.
- $\Delta \mu ^{(001)}_\text{KPZ}$ agrees with the crossover point between 2D single and poly-nucleation growth regime $\Delta \mu ^{(001)}_\text{poly}$, where $\Delta \mu ^{(001)}_\text{poly} = \Delta \mu ^{* (001)}_\text{poly} + c'/\ln (L/a)$.
- For $\Delta \mu ^{(001)}_{c, \text{MC}}$ $< \Delta \mu$, the surface grows via adhesive growth. The surface is atomically and thermodynamically rough. $\Delta \mu ^{(001)}_{c, \text{MC}}$ decreases as the temperature increases.
- For $\Delta \mu ^{(001)}_\text{BKT}< \Delta \mu < \Delta \mu ^{(001)}_\text{BtoK}$, the surface is BKT rough. $\Delta \mu ^{(001)}_\text{BKT}$ decreases as the temperature increases. Multilayered islands and negative islands on the terrace develop well to suppress the free step fluctuations on the surface.
- For $\Delta \mu ^{(001)}_\text{BtoK} < \Delta \mu$, the surface again tends to become KPZ rough. Similar to the case for the (2+1) Eden model, atoms on the surface detach rarely. $\Delta \mu ^{(001)}_\text{BtoK}$ slightly decreases as the temperature increases.

## Supplementary Information

Supplementary Information.

## Data Availability

The datasets used and/or analysed in the current study are available from the corresponding author upon reasonable request.
